# Polycythemia Vera Presenting With Normal Hemoglobin and Hematocrit: A Rare Variant

**DOI:** 10.7759/cureus.8404

**Published:** 2020-06-02

**Authors:** Burak Erdinc, Preethi Ramachandran, Avezbakiyev Boris

**Affiliations:** 1 Internal Medicine, Brookdale University Hospital Medical Center, Brooklyn, USA; 2 Oncology, Brookdale University Hospital and Medical Center, Brooklyn, USA; 3 Hematology/Oncology, Brookdale University Hospital and Medical Center, Brooklyn, USA

**Keywords:** jak2v617f mutation, masked polycythemia vera, portal vein thrombosis

## Abstract

Polycythemia vera (PV) is a myeloproliferative neoplasm, and its diagnosis requires elevated hemoglobin level (>16.5 mg/dL in men and >16 mg/dL in women), bone marrow characteristics of PV (hypercellularity for age with trilineage growth), and presence of JAK2 (Janus kinase 2) mutations or subnormal erythropoietin level if JAK2 mutation is not present. There exists a subset of patients with normal hemoglobin and hematocrit due to either from dilution of the blood or from coincidental blood loss anemia but these patients still might have underlying PV. These patients have masked PV, which is a variant of overt PV. We present a case of masked PV presenting with venous thrombosis as a first presentation and with normal blood counts. A 42-year-old male with past medical history of portal vein thrombosis and portal hypertension presented with nausea and vomiting presumably secondary to viral gastroenteritis. He was not an alcoholic nor a smoker. He was diagnosed with portal vein thrombosis six years ago which was treated with warfarin but was never investigated for a cause. His physical exam was within normal limits except he had splenomegaly. His laboratory values on admission showed hemoglobin of 14.1 g/dL, white blood count of 7.4 x10^9^/L, and platelet count of 164 x10^9^/L. His liver function test and renal function tests were within normal limits. His viral gastroenteritis improved within 48 hours. Extensive workup to rule out myeloproliferative neoplasm, thrombophilia, antiphospholipid syndrome, and paroxysmal nocturnal hemoglobinuria was arranged. Final results revealed JAK2V617F genetic mutation with a subsequent bone marrow analysis revealing a hypercellular marrow with increased trilineage hematopoiesis, consistent with primary PV. It is rare for myeloproliferative neoplasms to present with normal blood counts. There is a subgroup of patients with JAK2-positive PV presenting with normal hemoglobin and hematocrit. The prognosis of these subgroups seems to be poor especially when present in the older age group and with associated leukocytosis. Our case emphasizes two important points: first, need for extensive workup in a patient with unusual site thrombosis including JAK2 analysis and second, investigating for myeloproliferative neoplasm if presented with thrombosis even with normal blood counts.

## Introduction

Polycythemia vera (PV) is a disorder classified under myeloproliferative neoplasms (MPN) by the World Health Organization (WHO) along with essential thrombocythemia and primary myelofibrosis [1]⁠. PV is caused by stem-cell-derived clonal myeloproliferation which is characterized by JAK2 driver mutation 99% of the time, and almost all of the JAK2 mutations in PV are caused by the JAK2V617F gene located in exon 14 [2].

Diagnosis of PV requires elevated hemoglobin level (>16.5 mg/dL in men and >16 mg/dL in women), bone marrow characteristics of PV (hypercellularity for age with trilineage growth), and presence of JAK2 mutations or subnormal erythropoietin level if JAK2 mutation is not present. The disease can manifest with constitutional symptoms, such as fatigue and pruritus, hyperviscosity, leukocytosis, thrombocytosis or microcirculatory symptoms, and the course can be complicated with thrombosis, bleeding, leukemic transformation, or fibrotic progression [2]⁠.

There is a subset of patients with normal hemoglobin and hematocrit level but having all other criteria for diagnosis of PV (bone marrow findings, presence of JAK2 mutation, and subnormal serum erythropoietin level). These patients have masked PV (mPV), which is a variant of overt PV (oPV). We herein present a case of mPV initially presenting with portal vein thrombosis and was diagnosed with PV even though he had normal hemoglobin and hematocrit levels.

## Case presentation

Our patient is a 42-year-old male who initially presented to an outside hospital six years ago with hematemesis and subsequently diagnosed with upper gastrointestinal bleeding from esophageal varices and treated with endoscopic banding. At that time, he was diagnosed with portal hypertension secondary to acute portal vein thrombosis and received treatment with warfarin for six months and then took aspirin for an unknown period of time. Eventually, he stopped taking any medications and unfortunately lost follow-up due to lack of medical insurance. At the presentation to our hospital, he was not sure about his diagnosis and the reason why he had portal vein thrombosis. The patient denied any previous history of smoking tobacco products or using recreational drugs but reported drinking alcohol occasionally, not more than one or two drinks at a time. On the initial physical exam, his vital signs were within normal limits. The patient had a palpable spleen 2 cm below the left costal margin and reducible umbilical hernia. The rest of the physical exam was within normal limits. Initial laboratory investigations revealed white blood count of 7.4 x 10^9^/L with a left shift (93.2% neutrophils), hemoglobin level of 14.1 g/dL, platelet count of 138 x 10^9^/L, and normal kidney and liver function tests. His INR (international normalized ratio) was 1.45 without using any anticoagulation therapy. The patient also had mild anion gap acidosis (bicarbonate level of 20 mEq/L and anion gap of 14 mEq/L) with normal lactate level most likely secondary to acute gastroenteritis. His initial laboratory findings are summarized in Table 1.

**Table 1:** Initial Laboratory Investigations H: High, L: Low

	Results	Reference Range
White Blood Cell Count	7.4	4.10-10.10 × 10^9^/L
Red Blood Cell Count	4.52	4.33-5.43 × 10^12^/L
Hemoglobin	14.1	13.4-15.4 g/dL
Hematocrit	42.5	40.0%-47.0%
Mean Corpuscular Volume	94	80.8-94.1 fL
Platelet Count	138 (L)	153-328 × 10^9^/L
Neutrophils Absolute	6.90 (H)	1.40-6.80 × 10^9^/L
Lymphocytes Absolute	0.20 (L)	1.10-2.90 × 10^9^/L
Monocytes Absolute	0.3	0.20-1.00 × 10^9^/L
Eosinophils Absolute	0	0.00-0.40 × 10^9^/L
Basophils Absolute	0	0.00-0.10 × 10^9^/L
Prothrombin Time	16.5 (H)	9.2-12.8 sec
International Normalized Ratio	1.45 (H)	0.70-1.20
Partial Thromboplastin Time	27	23.5-35.5 sec
Glucose	131 (H)	70-99 mg/dL
Blood Urea Nitrogen	16	9.0-20.0 mg/dL
Creatinine	0.89	0.66-1.25 mg/dL
Sodium	140	133-145 mEq/L
Potassium	4.5	3.5-5.1 mEq/L
Chloride	106	98-107 mEq/L
Bicarbonate	20 (L)	22-30 mEq/L
Calcium	9.6	8.4-10.2 mg/dL
Anion Gap	14	8-12 mEq/L
Protein, Total	7.8	6.3-8.2 g/dL
Albumin	4.3	3.5-5.0 g/dL
Bilirubin, Total	3.6 (H)	0.2-1.3 mg/dL
Bilirubin, Direct	0.6 (H)	0.0-0.4 mg/dL
Alanine Transaminase	48	21-72 U/L
Aspartate Transaminase	50	17-59 U/L
Alkaline Phosphatase	60	38.0-126.0 U/L
Lipase	194	23-300 U/L
HIV AB 1 & 2	Negative	

**Figure 1:**
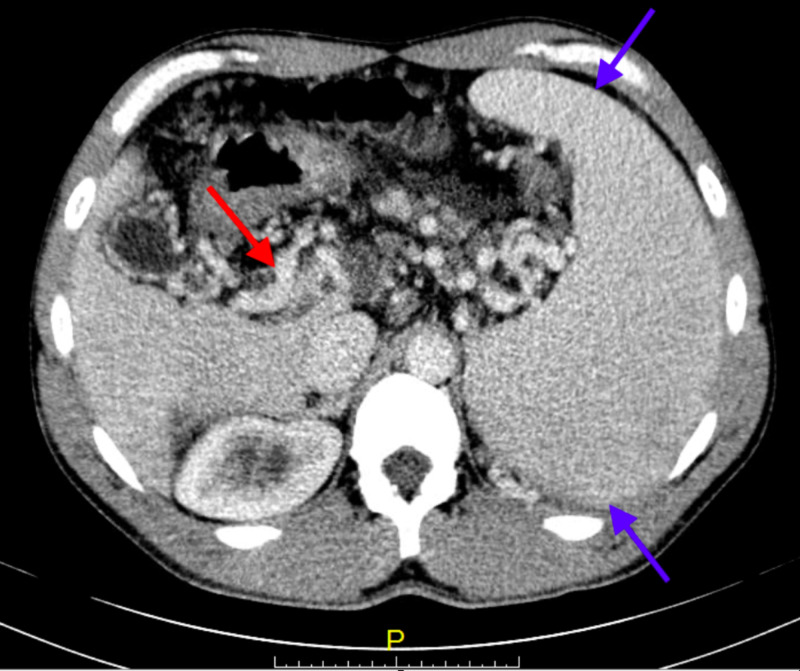
Cavernous transformation of the portal vein associated with chronic portal vein thrombosis (red arrow) and splenomegaly (blue arrows).

CT of the abdomen and pelvis with intravenous contrast showed mild intrahepatic biliary duct distention, chronic portal venous thrombosis, and cavernous transformation of the portal vein (Figure 1), extensive collateral vessels in the mesentery, mild ascites, splenomegaly (spleen size was 18.6 x 7.4 x 14.5 cm), and signs of acute enteritis and colitis. He was treated symptomatically for acute gastroenteritis most likely viral in nature due to the fact that stool studies showed no white blood cells, red blood cells, ova or parasites in microscopy exam, and the patient had negative stool culture.

**Figure 2:**
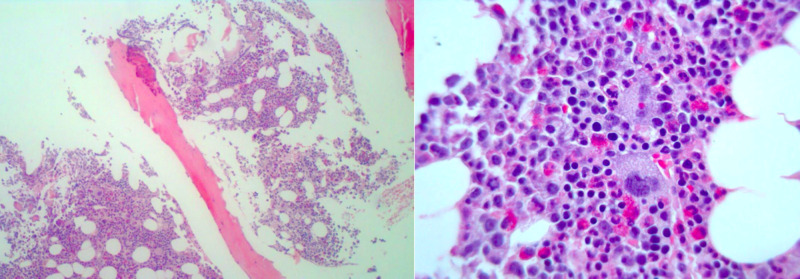
Low-power (left) and high-power (right) microscopy of the bone marrow: Hypercellular bone marrow with trilineage hematopoiesis and pleomorphic mature megakaryocytes consistent with polycythemia vera.

He had a bone marrow biopsy supporting a diagnosis of chronic MPN (Figure 2). There was hyperplastic marrow with increased trilineage hematopoiesis, bone marrow cellularity of 50%, 1% blast cells, adequate iron storage, and diffuse reticulin fibrosis (grade 0 of 3). There was evidence of increased numbers of megakaryocytic clusters with varying sizes and shapes. No increase in blasts or increase in reticulin fibers was noted. These changes were consistent with primary PV. Further workup revealed that the patient had JAK2V617F mutation with 15.25% JAK2 mosaicism.

The patient had an outpatient upper gastrointestinal endoscopy to assess for bleeding risk. It only showed two small columns of distal esophageal varices and no stigmata of recent bleeding. He eventually restarted apixaban therapy. While on oral anticoagulation, he had an episode of upper gastrointestinal bleeding and the endoscopy at the time of hospitalization revealed a non-bleeding duodenal ulcer with bleeding esophageal varices requiring banding. Therefore, he stopped taking anticoagulant therapy. This was complicated with acute pulmonary embolism, and the patient started on enoxaparin subcutaneous injections for anticoagulation. He has been tolerating low molecular weight heparin well, without any further episodes of bleeding. The patient will require long-term anticoagulant therapy. We will try switching to oral anticoagulant therapy later considering his risk of bleeding from the therapy. He recently started on pegylated interferon treatment to reduce thrombosis risk by reducing JAK2 allele burden. The patient received intravenous iron replacement therapy to treat iron deficiency anemia for a short period of time (ferritin level was 5.85 ng/mL) due to chronic blood loss and he has been in liver transplant list.

## Discussion

There are different cases of mPV with low hemoglobin level reported in the literature secondary to different etiologies, such as chronic blood loss due to bleeding from uterine fibroids and megaloblastic anemia due to previous duodenal ulcer treated with gastrojejunostomy [3,4]⁠. However, they all had a significant elevation in their hemoglobin level after the initial presentation⁠ as opposed to our case that our patient has not met the elevated hemoglobin/hematocrit criteria for two years which is most likely due to intermittent blood loss from bleeding esophageal varices. There is also a case of an mPV in the literature, which initially presented with extrahepatic portal venous obstruction but the patient was found to have high red cell mass in initial workup supporting the diagnosis as per WHO 2016 guidelines [5]⁠.

The most recent guidelines from World Health Organization (WHO) and British Criteria for Standards in Hematology (BCSH) now recommend red cell mass measurement as previously suggested by Tefferi et al. and McMullin et al. ​​*​*along with hemoglobin and hematocrit cut-offs as a major diagnostic criterion required for diagnosis of PV [6,7]⁠. Without measuring red cell mass, up to 45% of true PV patients used to be excluded by diagnostic criteria of PV according to Alvarez Larran et al. and Barbui et al. [8-10]⁠.

The term masked PV was suggested for the patients not meeting the cut-offs of hemoglobin and hematocrit levels as suggested in the WHO and BCSH guidelines but showing other diagnostic criteria, such as PV histology, JAK2 mutations, and subnormal erythropoietin levels [9,10]⁠. It is also shown that these patients carry a higher risk of developing the complications of transformation to myelofibrosis or acute leukemia and have a poorer overall prognosis [9,10]⁠. According to a study performed on 151 PV patients by Alvarez-Larran et al.⁠, patients with mPV were found to have higher platelet counts, lower JAK2V617F allele burden, and lower need for phlebotomies comparing to the oPV group [11]. They also showed faster response to cytoreduction therapy and higher complete hematological response rates [11]⁠.

## Conclusions

Physicians should be aware of clinical manifestations of PV, and extensive workup must be obtained including JAK2 analysis for patients with unusual site thrombosis such as portal vein thrombosis as in our case. Myeloproliferative disorders should be considered in the differential of this patient group even with normal blood counts.
